# Microfluidic Screening of Electric Fields for Electroporation

**DOI:** 10.1038/srep21238

**Published:** 2016-02-19

**Authors:** Paulo A. Garcia, Zhifei Ge, Jeffrey L. Moran, Cullen R. Buie

**Affiliations:** 1 Laboratory for Energy and Microsystems Innovation, Department of Mechanical Engineering, Massachusetts Institute of Technology, 77 Massachusetts Avenue, Cambridge, MA 02139 USA

## Abstract

Electroporation is commonly used to deliver molecules such as drugs, proteins, and/or DNA into cells, but the mechanism remains poorly understood. In this work a rapid microfluidic assay was developed to determine the critical electric field threshold required for inducing bacterial electroporation. The microfluidic device was designed to have a bilaterally converging channel to amplify the electric field to magnitudes sufficient to induce electroporation. The bacterial cells are introduced into the channel in the presence of SYTOX^®^, which fluorescently labels cells with compromised membranes. Upon delivery of an electric pulse, the cells fluoresce due to transmembrane influx of SYTOX^®^ after disruption of the cell membranes. We calculate the critical electric field by capturing the location within the channel of the increase in fluorescence intensity after electroporation. Bacterial strains with industrial and therapeutic relevance such as *Escherichia coli* BL21 (3.65 ± 0.09 kV/cm), *Corynebacterium glutamicum* (5.20 ± 0.20 kV/cm), and *Mycobacterium smegmatis* (5.56 ± 0.08 kV/cm) have been successfully characterized. Determining the critical electric field for electroporation facilitates the development of electroporation protocols that minimize Joule heating and maximize cell viability. This assay will ultimately enable the genetic transformation of bacteria and archaea considered intractable and difficult-to-transfect, while facilitating fundamental genetic studies on numerous diverse microbes.

Electroporation results from exposure of cells to external electric fields of sufficient strength to disrupt the plasma membrane^1^,^2^. The membrane disruption is attributed to the significant increase in local trans-membrane voltage (TMV) induced by the applied pulsed electric field^3^. When the TMV at a given point exceeds a critical threshold, the membrane is permeabilized and pores are created on the cell membrane, which mediate the transport of exogenous material into cells^4^. If the pores reseal, the electroporated cells can survive, and the process is termed reversible electroporation^3^. Otherwise, irreversible electroporation leads to cell death and is currently being evaluated as a new non-thermal tumor treatment^5^,^6^,^7^. Reversible electroporation is commonly used to deliver molecules such as drugs, proteins, or DNA into cells, but the mechanism remains poorly understood^8^,^9^,^10^,^11^. Understanding the electroporation process is critical for the burgeoning field of synthetic biology^12^ in which cells are programmed using foreign DNA to enhance their native capabilities, allow them to perform non-natural functions, and/or enable new applications in biotechnology such as production of alternative fuels^13^, enhancing oil recovery^14^, and even cancer treatment^15^.

There is a wealth of published electroporation protocols for genetic transformation of relevant strains for various biotechnology industries^16^. Despite the vast empirical literature establishing protocols for successful electroporation of cells, this process generally lacks real-time feedback for determining optimal conditions^17^. Previous studies have used 3D hydrogels and agar plates for characterizing the electric field threshold for irreversible electroporation (i.e. cell death)^18^,^19^. However, there are currently no protocols for determining the critical electric field for reversible (i.e. non-lethal) electroporation that do not rely upon time-consuming empirical experimental processes. Current state-of-the-art commercially available technologies for bacterial electroporation involve cuvettes containing planar electrodes separated by 1, 2, or 4 mm, which expose the cells to a uniform electric field. Therefore, experiments to determine the appropriate threshold for electroporation must involve multiple replicates at discrete electric field magnitudes. When performing reversible electroporation, it is important to apply the minimum electric field strength that will still induce electroporation, since this will mitigate Joule heating and associated cell death. Thus, there is a need for a rapid platform to systematically and quantitatively determine the critical electric field to enable delivery of external material into the cell.

Recent work on microfluidic electroporation has focused on the release of intracellular metabolites for analysis, delivery of exogenous agents for cellular manipulation in a flow-through manner, or manipulation at the single-cell level^20^,^21^,^22^. Wang *et al.* developed a microfluidic electroporation system based on geometric variations for electrical lysis of bacterial cells^23^. Geng *et al.* developed microfluidic chips with a series of constrictions with uniform cross-sectional areas for flow-through electroporation based on constant voltages^24^. Similarly, Adamo *et al.* developed a device with a comb electrode layout and characterized HeLa cell electroporation^25^. In terms of single-cell analysis, microhole structures in silicon nitride dielectric membranes have been used for trapping and electroporating single cells^26^. Similarly, Khine *et al.* developed a chip to selectively immobilize and locally electroporate single cells^27^. Finally, Boukany *et al.* developed a nanochannel to deliver transfection agents into living cells with electroporation^28^.

Complementary to the advances mentioned above, we have developed a microfluidic device to characterize the critical electric field for bacterial electroporation under specific experimental conditions (e.g. pulse duration, buffer conductivity, cell concentration) in a single experiment^3^. The microfluidic device consists of a bilaterally converging channel to amplify the electric field magnitude to sufficient levels to induce electroporation (Fig. 1). Additionally, the converging shape of the channel produces a spatially linear electric field gradient along its length^29^. Included in the channel with the cells is SYTOX^®^ Green nucleic acid stain (Life Technologies, Grand Island, NY), a fluorescent dye which shows a >500-fold fluorescence enhancement upon cytoplasmic nucleic acid binding^30^. The dye cannot penetrate the plasma membrane of living cells, but easily penetrates compromised plasma membranes, such as those induced by electroporation. Thus, the only cells in the channel that fluoresce are those which are exposed to an electric field strength greater than or equal to the critical electroporation threshold for the bacterium under investigation. Coupled with the linear electric field gradient, the dye allows for evaluation of the electric field strength required for electroporation without using discrete steps. Therefore, this microfluidic assay enables precise quantification of the critical electric field for electroporation in a single experiment, which would otherwise require hundreds of discrete experimental trials. Specifically, in our device we test the influence of electric field magnitude and cell type on electroporation. As test cases, we characterize the gram-positive bacteria *Corynebacterium glutamicum* (ATCC 13032, Manassas, VA, USA) and *Mycobacterium smegmatis* (ATCC, Manassas, VA, USA), and the gram-negative strain *Escherichia coli* BL21 (Bioline competent cells BIO-85032, London, UK). *C. glutamicum* has numerous industrial applications such as the production of enzymes, amino acids, and vitamins^31^. *M. smegmatis* is utilized in medical research as a model organism for disease-causing bacteria such as *M. tuberculosis*^32^. *E. coli* BL21 is a commonly used host for high-yield expression of recombinant proteins in biological studies^33^.

## Results

### Microfluidic Electroporation Assay with Linear Electric Field Gradient

**Figure 1:**
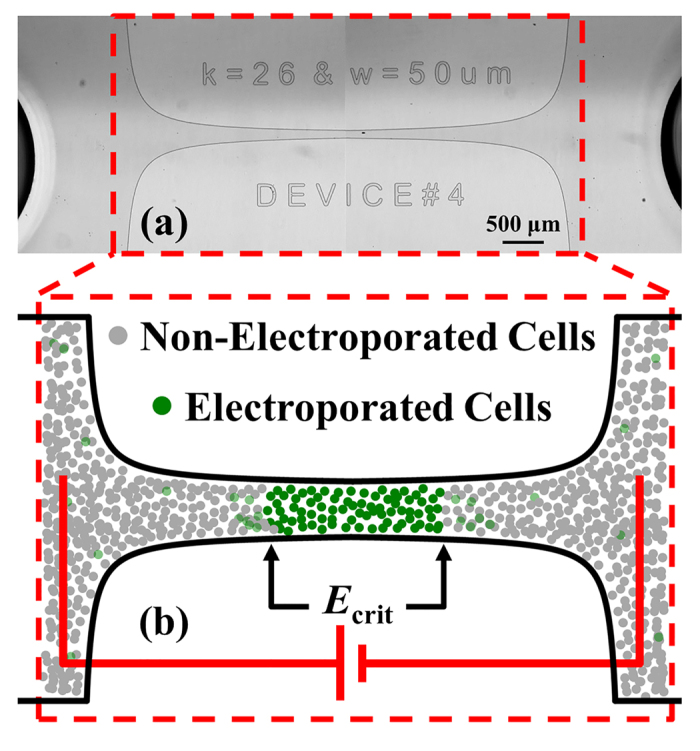
Microfluidic device to determine the critical electric field required for bacterial electroporation. (**a**) Two adjacent microphotographs showing the entire bilaterally converging channel (red-dashed outline) that amplifies the electric field to levels necessary to induce bacterial electroporation. (**b**) Schematic representation of the magnified constriction region illustrates the increase in green fluorescence due to SYTOX^®^ dye uptake after electroporation with electric fields *E *≥ *E*_*crit*_. (panel (**b**) not drawn to scale).

Figure 1 shows the microfluidic device used to determine the critical electric field from a population of cells based on their response to pulsed electric field exposure. Upon application of a voltage along the device, the electric field within the microfluidic device exhibits a linear gradient, allowing cells to experience location-dependent electric field strengths within the channel. The cells are introduced with the nucleic acid stain SYTOX^®^ Green into the channel. SYTOX^®^ permeates the cell membranes and fluorescence is enhanced >500-fold in the cells that experience *E *≥ *E*_*crit*_, where *E*_*crit*_ is the critical electric field strength to cause electroporation of the cell. The location of the transition between electroporated and non-electroporated cells can be determined from fluorescent images, and enables facile determination of the critical electric field threshold.

**Figure 2:**
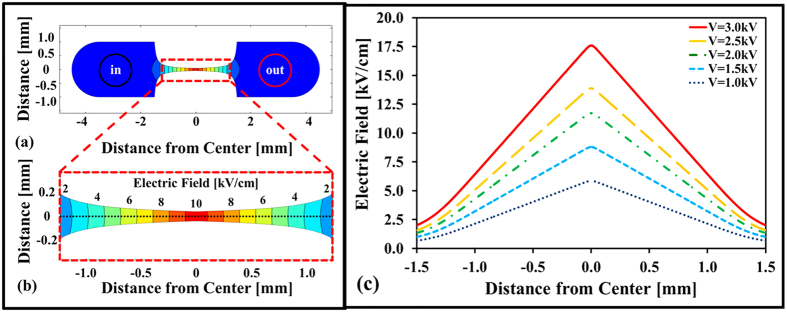
Representative electric field distribution for conducting electroporation assay in microfluidic device. Microfluidic device (**a,b**) demonstrating the 3-mm constriction that amplifies the electric field to magnitudes of sufficient strength to induce electroporation. The device constriction produces (**c**) a linear electric field gradient along the black-dotted line in panel (**b**) that can be spatially correlated with the location of the fluorescence enhancement.

The electric field gradient is a function of the applied voltage and the channel geometry. Figure 2 shows the channel with a linear electric field gradient along the 3-mm constriction length. Figure 2a,b shows the electric field distribution within the channel. Additionally, by varying the applied voltage one can broaden or compress the range of electric fields applied to the cells (Fig. 2c). The converging geometry in the device results in a linear electric field gradient, as can be seen in Fig. 2c. Due to the symmetrical geometry of the channel, two *E_crit_* measurements are possible from a single experiment. The bilaterally converging geometries can be tuned to have steeper or shallower electric field gradients by modifying the constriction ratio and adjusting the channel length.

In reversible (transient) electroporation, maintaining high cell viability is a pre-requisite for protein expression. The goal of the microfluidic assay is to determine the critical electric field for the onset of electroporation. Thus, we determine the location of the transition between non-electroporated and electroporated cells, which occurs at significantly lower electric fields than irreversible electroporation (i.e., cell death). In order to maintain high cell viability, we designed the microfluidic device to achieve maximum electric field strengths of approximately 15 kV/cm at an applied voltage of 2.5 kV. This ensured that the electric field range evaluated was below the 15 kV/cm experimental limit in which *E. coli* viability is compromised after exposure to a 1.0 ms pulsed electric field^34^. Exposure of cells to stronger electric fields, longer pulse duration, or even a larger number of pulses will have a negative impact on cell viability^34^,^35^. Therefore, the present assay was limited to a single 1.0-ms electric pulse to establish the lowest electric field required for electroporation while preserving high cell viability.

### Representative Fluorescent Images for Electroporation Assay

**Figure 3:**
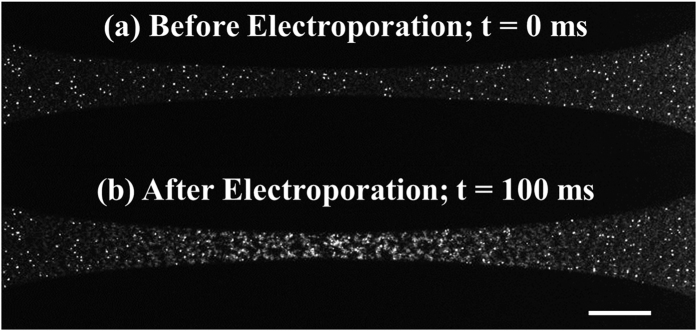
Fluorescent images for detection of boundary between electroporated and non-electroporated bacteria. Fluorescent images (**a**) before and (**b**) after delivering a 1.8-kV exponentially decaying (*t* = 1.0 ms; *τ* = 5.0 ms) pulse in 0.01× phosphate buffered saline (PBS) buffer (PBS diluted 100 times in DI water) and 5 μM SYTOX^®^ Green nucleic acid stain to *C. glutamicum* bacteria (scale bar = 200 μm).

The critical electric field is quantified by analyzing fluorescent images captured before and after electric pulsing. Figure 3 displays fluorescent images of *C. glutamicum* before and after a truncated (*t* = 1.0 ms) 1.8-kV exponentially decaying pulse (with decay constant *τ* = 5 ms) was delivered. Prior to pulse delivery, some background fluorescence was detected (Fig. 3a). The background fluorescence is proportional to the number of dead or already-compromised cells in the channel. Figure 3b shows fluorescence detected 100 ms after pulse delivery, in which the fluorescence is qualitatively enhanced compared to Fig. 3a. The representative panels provide the raw data used during image processing to correlate the location of fluorescence enhancement with the simulated electric field distribution (Fig. 2). The fluorescent images demonstrate that electroporation can be induced and detected in our microfluidic device, sampling a continuum of electric field strengths in a single experiment.

### Image Processing Methodology

**Figure 4:**
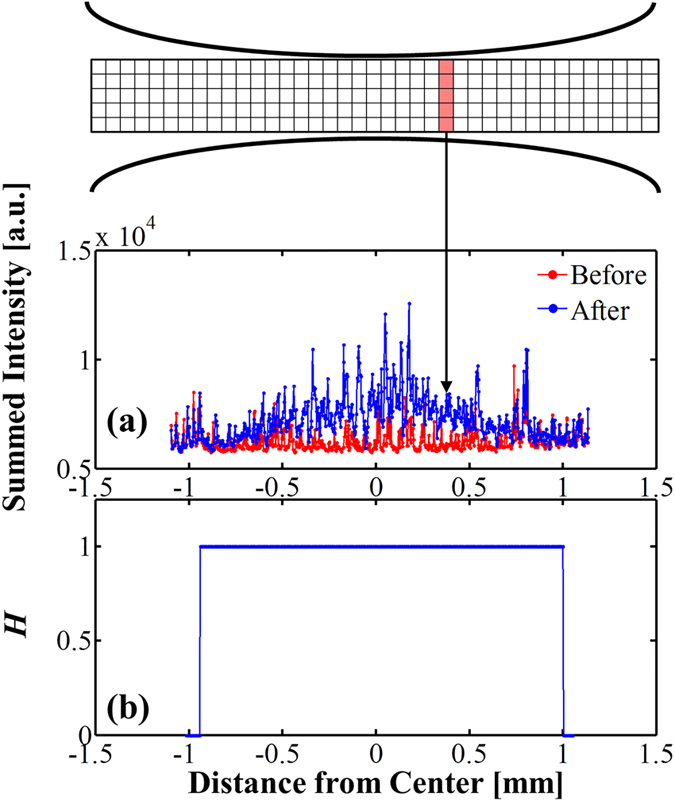
(**a**) Summed intensity (a.u., arbitrary units) versus distance from the channel center. The summed intensity at each point is equal to the sum of the intensity values in the (shaded) vertical column of pixels located at the point of interest. (**b**) Result of the one-sided two-sample Kolmogorov-Smirnov (KS) test as a function of position along the channel. The value of *H* at a given location indicates that the null hypothesis *H_0 _* was either accepted (*H* = 0) or rejected (*H* = 1) for a 51-pixel stencil centered at that location. As discussed in the main text, *H* = 1 for regions in which a significant portion of the post-pulse fluorescence intensity values exceed the pre-pulse values. In general, the location at which *H* changes from 0 to 1 is taken as the location of onset of fluorescence enhancement due to electroporation. For the case depicted above (*C. glutamicum*, *V_app_* = 1.8 kV), the predicted values of the critical electric field (found by linearly interpolating the simulated electric field data visualized in Fig. 2a,b) are 4.28 kV/cm (left) and 3.85 kV/cm (right), yielding an average *E*_*crit*_ = 4.07 kV/cm. Note that in cases such as this, in which the entire channel is in the microscope’s field of view, it is possible to exploit the bilateral symmetry of the channel geometry and obtain two estimates of *E*_*crit*_ – one on each end of the channel.

Although the increase in fluorescence due to dye uptake by electroporated cells is often easy to locate visibly, the precise location of the onset of fluorescence can be more accurately quantified with image analysis. Figure 4a shows the summed fluorescence intensity (defined as the sum of the intensities in the shaded pixels) as a function of position along the channel. The red “Before” data is taken from the last image before the pulse is applied (the same image shown in Fig. 3a) and the blue “After” data is captured 200 ms after the pulse. In most cases, this slight delay allows the cells to fully uptake the dye and reach their steady-state post-pulse fluorescence intensity. In some cases dye uptake requires more time than 200 ms; in these cases the “After” data is captured 1 s after the pulse. These exceptions are indicated with an asterisk in Table 1.

The critical electric field is defined as the electric field magnitude at the location of the onset of electroporation-induced fluorescence enhancement. To quantitatively estimate this location, we use the two-sample Kolmogorov-Smirnov (KS) test, a well-established statistical procedure for determining whether or not two data sets are drawn from the same underlying probability distribution^36^. The KS test considers the null hypothesis that two discrete datasets are drawn from the same (unknown) continuous probability distribution against a user-specified alternative hypothesis. The standard two-sided KS test considers the alternative hypothesis that the distribution functions of the two datasets are unequal, without regard to which is larger or smaller. However, in this work we are specifically interested in finding the regions of the channel where the post-pulse fluorescence intensity *exceeds* the pre-pulse intensity. Therefore, we use a *one-sided* KS test which evaluates the null hypothesis defined above (that both datasets are drawn from the same distribution) against the alternative hypothesis that the cumulative distribution function underlying the pre-pulse dataset is *larger* than that of the post-pulse dataset (as opposed to the two being simply unequal). Defined this way, the KS test will reject the null hypothesis in favor of the alternative hypothesis in regions of the channel where the post-pulse intensity significantly exceeds the pre-pulse intensity.

Consider two sets of fluorescence intensity values, *I*_*before*_ and *I*_*after*_, which respectively represent the sets of intensity values before and after the pulse for a given sub-region *R* of the channel. From these datasets one can construct empirical cumulative distribution functions (CDFs), *S*_*before*_ and *S*_*after*_. Then let *F*_*before*_ and *F*_*after*_ be the corresponding true (but unknown) population cumulative distribution functions for *I*_*before*_ and *I*_*after*_. Both *F*_*before*_ and *F*_*after*_ are normalized, so that both 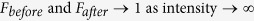. The null hypothesis *H*_0_ to be tested is that the underlying distribution functions are identical, i.e. that both datasets are drawn from the same distribution:


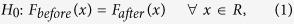


where *x* is the distance along the channel constriction and *R* is the sub-region of the channel under consideration. In the regions of the channel where the electric field is insufficient to induce electroporation, there should be a negligible difference between the pre- and post-pulse intensity data. In these regions, the KS test should fail to reject the null hypothesis (i.e., in these cases we would expect the KS test to conclude that the two datasets are indeed drawn from the same distribution).

However, as the region *R* moves toward areas of increasing electric field strength, eventually the post-pulse data will deviate from the pre-pulse data due to electroporation-induced fluorescence enhancement in the post-pulse data. If enough of this data is included in *R*, the null hypothesis will be rejected in favor of the one-sided alternative hypothesis *H*_1_:


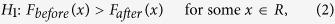


We note that, somewhat counterintuitively, if 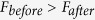, this implies that a significant portion of the data points in the *pre-pulse* data (captured before the pulse) have a *lower* intensity. Thus, we should expect that *H*_1_ will be favored for regions *R* where the post-pulse fluorescence is significantly enhanced due to electroporation.

Here, we perform the KS test sequentially for a 51-pixel stencil (which constitutes the region *R*) that begins at one end of the channel and moves horizontally along the channel, one pixel at a time. At each stencil location, the KS test is performed for the pre-pulse and post-pulse intensity values (shown in Fig. 4a) for the 51 adjacent pixels, and a value *H* is recorded at the pixel occupying the center of the stencil (see Fig. 4b). The binary parameter *H* is defined as follows:


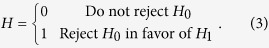


In words, we expect that *H* = 1 in regions where electroporation has occurred and *H* = 0 in regions where electroporation has not occurred.

Finally, to estimate the critical electric field, for each dataset we examine the locations where *H* = 1, and among these points, find the location where the electric field is minimized. This location is taken as the location of the onset of electroporation, and the electric field at this location (calculated by linear interpolation of the simulation data displayed in Fig. 2c) is taken as the minimum electric field for electroporation for the given experimental trial.

Figure 4b shows the KS test result *H* as a function of position for an example case of *C. glutamicum* exposed to a 1.8-kV 1-ms pulse. For this particular case, the critical electric field for electroporation is estimated as 4.07 kV/cm. Note that here the KS test is able to identify two transition zones, one on each end of the channel. This enables one to exploit the bilateral symmetry of the channel geometry to obtain two estimates of *E*_*crit*_ in one experiment. The value 4.07 kV/cm is the average of the KS-test-determined values on each end. Table 1 shows quantitative estimates of the critical electric field for electroporation of *C. glutamicum*, *M. smegmatis*, and *E. coli* BL21 using the method described above. Note that all of the data displayed in Table 1 consists of a single estimate for each experiment; in these cases, the entire channel was not within the microscope’s field of view, and so the KS test was only able to detect one transition.

### Critical Electric Field Threshold for Electroporation

**Figure 5:**
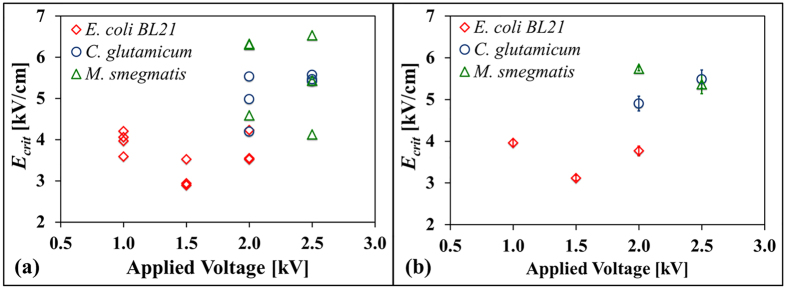
Critical electric field (*E*_*crit*_) for bacterial electroporation as a function of applied voltage. Panel (**a**) shows the values obtained from individual experiments, visualizing the data shown in Table 1; panel (**b**) shows averages (error bars show ± **Δ*****E***_**crit**_) for each bacterium at each applied voltage. These values indicate that (gram-negative) *E. coli* BL21 requires a smaller *E*_*crit*_ than *C. glutamicum* and *M. smegmatis* (gram-positive) bacteria.

Both gram-positive (*C. glutamicum* and *M. smegmatis*) and gram-negative (*E. coli* BL21) strains have been tested in the microfluidic device. Figure 5 plots the electric field thresholds for electroporation of *C. glutamicum* (5.20 ± 0.20 kV/cm @ 2 kV and 2.5 kV), *M. smegmatis* (5.56 ± 0.08 kV/cm @ 2 kV and 2.5 kV), and *E. coli* BL21 (3.65 ± 0.09 kV/cm @ 1.0 kV, 1.5 kV, and 2 kV). The shift of cells during and after the pulse was quantified by tracking the motion of a single fluorescent bacterium at the transition zone and correlating the distance traveled by this bacterium from the last pre-pulse to the first post-pulse image to the corresponding shift in local electric field experienced by that bacterium. The induced shift could be generated by electrophoresis, electroosmotic flow, and/or pressure gradients. This shift in electric field is reported as the uncertainty measurement (Δ*E*_crit_) for each experiment conducted. The average in the thresholds was calculated from the replicates of each strain across all applied voltages. The average calculation across all applied voltages is appropriate since electroporation is an electric-field-dependent physical phenomenon. Consistent with published protocols in the literature, we confirm that *E. coli* BL21 (gram-negative) requires a weaker electric field to induce electroporation than both gram-positive bacteria studied, suggesting that membrane composition is a potential contributor to the electroporation outcome^5^.

## Discussion and Conclusions

The electroporation assay developed in this work correlates the location where fluorescence is significantly enhanced with the computed electric field. The efficacy of the method is vulnerable to flow in the channel during imaging and to the sensitivity of the microscope to detect fluorescence. To mitigate the influence of flow on *E*_*crit*_ measurement, we balanced the pressure of the inlet and outlet by using tubing clamps and waiting until flow subsided before applying the electric pulse. The electric pulses employed for electroporation also induce some electrophoretic motion of the cells and electroosmotic flow during pulse delivery. However, our experimental protocol employed truncated pulse durations of 1.0-ms that induce a relatively small shift (Table 1), as can be verified with the sequential images in Fig. 3 and slight asymmetry in the left and right transition locations, as shown in Fig. 4b. More importantly, we analyzed multiple frames after the initial pulse in order to allow for the fluorescence enhancement to proceed to completion. We mitigate the sensitivity limitations of the camera by using relatively long exposure times of 100 ms.

Since the TMV distribution of a cell under an applied electric field may be influenced by the presence of nearby cells as demonstrated by Kotnik *et al.*^37^, it is possible that in our experiments the calculated value of *E*_*crit*_ could depend on the cell concentration. However, we argue below that this effect is negligible for the conditions considered in this work and that the *E*_*crit*_ values obtained are applicable to individual cells.

The induced transmembrane voltage for spherical cells in close proximity was studied by Kotnik *et al.*^38^, who considered both solitary cells and dense cell suspensions (up to 50% volume fraction). For a solitary cell under an applied electric field *E*, it is well known that the TMV (written here as *V*_*m*_) can be computed analytically using the steady-state Schwan equation:


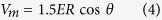


where *R* is the cell radius and the angle *θ* is measured from the center of the cell with respect to the electric field. Kotnik *et al.* compared this analytical result to numerical computations of the TMV for spherical cells arranged in a face-centered cubic (FCC) lattice, as a model of highly concentrated cell suspensions. They show in their Fig. 4 that the TMV varies by a maximum of ~15% for cell volume fractions ranging from 10% to 50%. In our experiments, we estimate the cell volume fraction to be ~ 0.7% by assuming a single cylindrical *E. coli* bacterium with length 2 μm and diameter 1 μm (Volume = 1.57 × 10^−12^ mL). A cell concentration of 3.85 × 10^9 ^cells/mL would then occupy a volume of 0.00685 mL (0.685%), which is well below the cell volume fractions considered by Kotnik *et al.* Therefore, the cell volume fraction is low enough for the induced TMV distribution (and, by extension, the calculated value of *E*_*crit*_) to vary negligibly from the corresponding values for single cells. We conclude that neighboring cells do not significantly influence the calculated values of *E*_*crit*_ for the experiments in this work.

The microfluidic assay presented here has several key advantages compared to the traditional trial-and-error approach for determination of electric fields for electroporation. First, the assay allows one to sample a continuum of electric field magnitudes in a single experiment. This facilitates the quantitative determination of the critical electric field, greatly reducing the number of experiments required and promoting electroporation conditions that maximize cell viability. Second, the assay uses a minimal amount of sample (~0.2 μL), compared to traditional electroporation cuvettes that require 100–200 μL of sample. Thus, a larger number of experiments (by several orders of magnitude) can be performed with the same volume of sample. Lastly, the assay is independent of growth conditions. Optical access to the electroporation experiment allows for near real-time evaluation of electroporation conditions.

The microfluidic assay enables rapid quantification of the critical electric field for electroporation in a single experiment. This requires the presence of the fluorescent dye in the sample prior to the delivery of the electric pulses in order to achieve responses within milliseconds. However, having the SYTOX^®^ Green dye present prior to pulsing does not allow for discrimination between cells that have undergone reversible electroporation (transient) and those that have undergone irreversible electroporation (permanent) as both become labeled simultaneously. However, we do not expect major changes in cell viability since the electric field and pulse duration used is within the sub-lethal limits identified in Alvarez *et al.* in other studies^34^. Future work will devise a microfluidic assay that will enable quantification of the minimum and maximum electric fields for successful transformation. To verify both successful transformation and cell viability, we plan to immobilize cells and culture for several hours post-electroporation to detect protein expression. This will allow for varying other important parameters during electroporation such as pulse number, pulse duration, and delays between pulses.

The microfluidic device allows electroporation within the constriction region by exposing cells to a continuous electric field spectrum. However, there can be therapeutic or biotechnology applications in which it is desired to expose cells to a broader spectrum of continuous electric fields. In this case, a cell suspension may be driven through the channel at a constant volumetric flow rate; the cells may then be exposed to a constant applied voltage (but time-dependent electric field) for the entirety of their residence in the channel. This would be a new flow-through transformation platform, similar to the device of Lu and co-workers^24^,^39^, that would eliminate the electric field as a variable but would require coordination between pulse delivery and volumetric flow rate to avoid exposing cells to multiple pulses that could reduce cell viability.

In summary, we have developed a rapid microfluidic assay to test a continuum of electric field strengths in a single experiment. The assay allows for real time, optical evaluation of the effect of electric field on pore formation. The use of a constricted channel enables the quantitative evaluation of the critical electric field that results in pore formation and transfection. We apply the assay to a diverse array of gram-positive and gram-negative bacteria in order to demonstrate strain-specific determination of the critical electric field for electroporation. The assay has thus far been applied exclusively to bacteria but could also be utilized for eukaryotes and archaea. In the future we envision this assay as a useful tool to devise electroporation protocols for microbes previously considered difficult-to-transfect or intractable.

## Materials and Methods

### Bilaterally Converging Channel Geometry

A mathematical function was used to define the constriction geometry. The curve defining the channel width is given by:


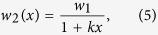


where *w*_1_ is half-maximum channel width and *w*_2_ is the half-width as a function of distance *x* along the length of the constriction region^29^. The parameter *k* defines the degree of tapering between the maximum and minimum dimensions within the constriction region. The parameter values for the experimental device are *w*_1_ = 1 mm, *k* = 26 mm^−1^, which corresponds to 0 < *x *< 1.5 mm. The curve may then be reflected about the *x* = 1.5 mm plane in addition to the *y* = 0 plane in order to generate a symmetric volume with a total constriction region length of 3.0 mm. This specific combination of parameters results in a minimum constriction width of 50 μm. Finally, the device was extruded by 15 μm in the *z*-direction to generate the 3D volume used during the experiments to allow for focusing of the cells without excessive background interference. Modifying the geometrical combination of channel parameters may be used to increase or decrease the constriction region length and control the level of electric field amplification.

### Microfabrication Protocol of Devices

The photomasks were designed in AutoCAD 2014 (Autodesk, San Rafael, CA) with the desired geometrical features optimized using computational models (COMSOL Multiphysics, Burlington, MA) and printed by a commercial vendor (Fine-Line Imaging, Colorado Springs, CO). The devices were fabricated using traditional soft lithography techniques^40^. The features were patterned on a silicon wafer using SU-8 2015 (Micro-Chem, Westborough, MA) Permanent Epoxy Negative Photoresist after single-chamber (4″ barrel asher) O_2_ plasma exposure for 5 min (200 W and 0.5 Torr), followed by dehydration at 200 °C for 10 min. A spin speed of 3000 rpm was used during the coating step to achieve a channel height of 15 μm, in order to focus the field of view relatively close to the bacteria dimensions (~2 μm). Then, the silicon wafer was soft baked on a hot plate and exposed for 1 min at 65 °C followed by 3 min at 95 °C. The SU-8 was then cured by exposing to two 14 s UV cycles separated by a 14 s interval at a rate of 10.5 mW/s. The post exposure bake used a 2 min cycle at 65 °C followed by a 4 min cycle at 95 °C. Next, the wafer was exposed to a developer (PM Acetate) at a spin speed of 500 rpm which dissolved the regions not exposed to the UV and was rinsed with Isopropyl Alcohol. Once the master was fabricated, the surface was treated under vacuum for at least 2 hours with tridecafluoro-1,1,2,2-tetrahydrooctyl-1-trichlorosilane (Sigma Aldrich, St. Louis, MO) for easier release. Then, PDMS Sylgard 184 (Dow Corning, Midland, MI) was used at a 10:1 ratio and placed under vacuum for at least 2 hours to remove any bubbles from the polymer. The master and PDMS were then placed inside an oven to cure overnight at 75 °C. Once the devices were ready to be fabricated, each microdevice was isolated from the master with an X-Acto knife (Elmer’s Products, Westerville, OH) and washed and rinsed with acetone, isopropyl alcohol, and DI water and air dried with compressed gas. The glass slides were also washed and rinsed with the same protocol. The PDMS devices were bonded to a glass slide after a 45 s plasma treatment and placed in oven at 75 °C overnight prior to experimentation.

### Culturing Conditions for Bacterial Strains

Bacterial strains were cultured overnight in a 3-mL test tube of Luria broth (LB) medium (*E. coli* BL21), 7H9 supplemented with OADC and Tween-80 (*M. smegmatis*) or brain heart infusion supplemented with 0.5 M sucrose (BHIS) medium (*C. glutamicum*). The following morning, 333 μL of cell culture was transferred to 50 mL of fresh growth media and allowed to grow to exponential phase before the electroporation assay (OD_600 _between 0.5–0.8). Each of the strains studied were concentrated 9 times by centrifuging at 8000 rpm. The supernatant was discarded and the cells were washed two additional times with 0.01× PBS supplemented with 0.1% (v/v) Tween 20 in order to avoid agglomeration of the cells. Cells were then re-suspended in 0.01× PBS buffer. Immediately prior to the assay, 5 mM SYTOX^®^ dye was added to the cell solution for a final concentration of 5 μM in all the experiments.

### Electroporation Protocol and Image Data Collection

The cells were introduced into the microfluidic devices at a flow rate of 50 μL/min until there was visible solution at the outlet. This indicates that the constriction region is filled and that there is good electrical contact with the platinum electrodes connected to the Micropulser^TM^ Electroporator (Bio-Rad, Hercules, CA). The inlet tubing was then clamped with hemostats to mitigate undesired flow. Once the flow is stopped, the data collection is started in the form of fluorescent images using the green fluorescence filter (Nikon 96311 B-2E/C) in the microscope. The microscope was programmed to collect images every 100 ms and had exposure times of 100 ms as well. A total of 200 images were captured for each of the twenty-two experimental trials. The cells were exposed to a single exponentially decaying (duration *t* = 1.0 ms; decay constant *τ* = 5 ms) pulse with applied voltages of 1.0 kV, 1.5 kV, 2.0 kV, or 2.5 kV.

### Image-processing Methodology of Fluorescent Images

**Figure 6:**
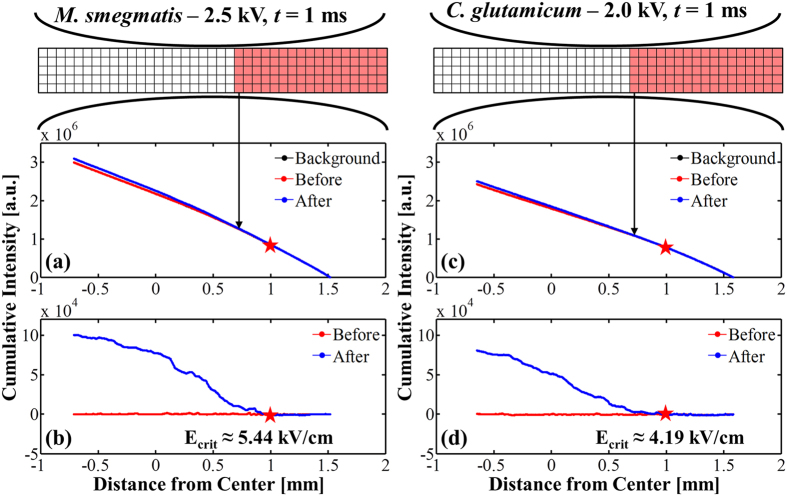
Image-processing methodology to compute the cumulative fluorescence intensity along the channel and enable rapid visual identification of the location of electroporated cells. (**a**) The cumulative intensity *CI* at a point (indicated by the black arrow) is defined as the sum of the red shaded pixel values. In this figure, the black “Background” curve is virtually indistinguishable from the red “Before” curve. (**b**) The adjusted cumulative intensity *CI’* is the same as in (**a**) but with the background subtracted to accurately determine the location of the onset of electroporation along the channel, which is indicated with a red star in both subfigures. This figure allows visual identification of the onset of electroporation-induced fluorescence enhancement, and agrees with the quantitative findings of the KS test. Panels (**c**,**d**) are analogous to (**a,b**) for a case of *C. glutamicum* subjected to a 1-ms 2-kV voltage pulse.

Fluorescent images are collected before, during, and after the delivery of the electric pulse. In regions where the electric field is strong enough to disrupt the cell envelope, the SYTOX^®^ dye permeates the membrane, binds to cytoplasmic DNA, and the resulting fluorescence is enhanced above the natural background intensity. The variation in fluorescence along the channel is analyzed quantitatively using a custom MATLAB program (MathWorks, Natick, MA, USA) to determine the location of this transition (Fig. 6). The electric field at the transition location is determined from computational simulations in COMSOL Multiphysics (COMSOL, Inc., Burlington, MA, USA).

To visualize the location of the critical electric field magnitude for electroporation, it is useful to define the cumulative fluorescence intensity, *CI*, in terms of the intensity value assigned to a given pixel, *I*. Here, we define *CI* at a given point *x*_*n*_ along the channel as the sum of the intensity values for each pixel in the vertical direction (over all values of *j*), for all columns to the right of and including *x*_*n*_. Mathematically,


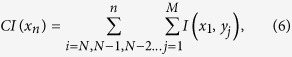


where *I* is the intensity of a given pixel in arbitrary units. The summation is conceptually illustrated in Fig. 6 above; note that the summation is carried out from right to left (i.e., the summation index *i decreases* from *N* to *n*). The channel is spatially discretized into an array of square pixels, *N* = 696 pixels along the length by *M* = 17 pixels along the width of the channel.

To enable rapid visual identification of the location of the onset of fluorescence enhancement, we also computed the adjusted cumulative fluorescence intensity, *CI’*. To compute *CI’*, we subtract the cumulative intensity *CI*_*back*_ of the average of the 5 images directly before the pulse from the pre- and post-pulse images given by:


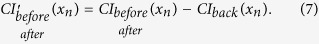


Figure 6 shows the image-processing methodology for computing both the fluorescence intensity (*CI*) (middle) and adjusted cumulative fluorescence intensity (*CI’*) (bottom) for *C. glutamicum* and *M. smegmatis*. Plotting the adjusted cumulative fluorescence intensity enables rapid estimation of the location in the channel at which fluorescence enhancement begins, enabling calculation of the approximate electric field threshold for a given bacterial cell type. However, we emphasize that the critical electric field values listed in Table 1 and plotted in Fig. 5 are computed from the more quantitative KS test described above, not from the cumulative intensity plots. The plots in Fig. 6 are intended as visual confirmation of the values calculated using the KS test.

Figure 6 demonstrates the methodology for computing the cumulative intensity vs. position from the raw fluorescence data along the channel. The cumulative intensity at a point represents the sum of the intensity values in all of the shaded pixels (Fig. 6a-6c). The red and blue curves represent the cumulative intensity vs. position for the last image captured before the pulse and first image captured after the pulse, respectively. The black curve (nearly indistinguishable from the red curve) represents the average cumulative intensity for 5 images captured prior to the red curve. Figure 6b-6d show the same data plotted in panel 6a and 6c with the black curve subtracted from both the blue and red curves respectively. This subtraction makes clear the location of the onset of fluorescence due to permeabilization of the bacterial membranes and the resulting uptake and fluorescence of SYTOX^®^, which allows for easy correlation with the electric field data from the computational models.

## Additional Information

**How to cite this article**: Garcia, P. A. *et al.* Microfluidic Screening of Electric Fields for Electroporation. *Sci. Rep.* **6**, 21238; doi: 10.1038/srep21238 (2016).

## Figures and Tables

**Table 1:** Estimates of critical electric field [kV/cm] magnitude (±Δ*E*_crit _) for electroporation of *C. glutamicum*, *M. smegmatis,* and *E. coli* BL21 exposed to a single exponentially decaying (*t* = 1.0 ms; *τ* = 5.0 ms) pulse in 0.01× PBS buffer with 5 μM SYTOX^®^ Green nucleic acid stain.

Trial	V_*applied*_ [kV]	*C. glutamicum*		*M. smegmatis*		*E. coli* BL21	
		*E*_crit_	Δ*E*_crit_	*E*_crit_	Δ*E*_crit_	*E*_crit_	Δ*E*_crit_
1	1.0	–	–	–	–	4.06	0.03^#^
2	1.0	–	–	–	–	4.21	0.08
3	1.0	–	–	–	–	3.59	0.15
4	1.0	–	–	–	–	3.97	0.06
5	1.5	–	–	–	–	2.89	0.09
6	1.5	–	–	–	–	2.94	0.06
7	1.5	–	–	–	–	3.52	0.03
8	2.0	5.53	0.21	4.59	0.03	3.52	0.10
9	2.0	4.19	0.16	6.33	0.05	4.23	0.19
10	2.0	4.98	0.16	^*^6.31	0.14	3.55	0.07
11	2.5	5.47	0.15	5.44	0.11	–	–
12	2.5	5.58	0.32	^*^4.13	0.12	–	–
13	2.5	5.42	0.19	6.53	0.05^#^	–	–
Average	–	5.20	0.20	5.56	0.08	3.65	0.09

^#^Denotes a left shift between t = 0 and t = 200 ms. Right shift for all other cases.

The critical electric field is defined as the magnitude of the electric field at the location of the onset of electroporation. This location is estimated using the one-tailed two-sample Kolmogorov-Smirnov test^36^, and is defined as the electric field at the location where the KS test first determines that the pre-pulse and post-pulse data sets are from different underlying probability distributions. A 5% significance level is used in all cases (*α* = 0.05). NOTE: A ‘*’ indicates that the pre-pulse and post-pulse images are collected at 1 s intervals instead of the usual 200 ms, due to slower uptake of the dye in these cases.
